# Techno-Functional and Nutraceutical Assessment of Unprocessed and Germinated Amaranth Flours and Hydrolysates: Impact of the Reduction of Hydrolysis Time

**DOI:** 10.3390/foods14152666

**Published:** 2025-07-29

**Authors:** Alvaro Montoya-Rodríguez, Maribel Domínguez-Rodríguez, Eslim Sugey Sandoval-Sicairos, Evelia Maria Milán-Noris, Jorge Milán-Carrillo, Ada Keila Milán-Noris

**Affiliations:** 1 Laboratorio de Nutracéuticos (#18), Facultad de Ciencias Químico Biológicas, Universidad Autónoma de Sinaloa, Calzde las Américas Nte 2771, Cd Universitaria, Burócrata, Culiacán 80030, Sinaloa, Mexicosugeysandoval@uas.edu.mx (E.S.S.-S.);; 2 Programa de Posgrado Integral en Biotecnología, Facultad de Ciencias Químico Biológicas, Universidad Autónoma de Sinaloa, Calzde las Américas Nte 2771, Cd Universitaria, Burócrata, Culiacán 80030, Sinaloa, Mexico; 3 Posgrado en Ciencia y Tecnología de Alimentos, Facultad de Ciencias Químico Biológicas, Universidad Autónoma de Sinaloa, Calzde las Américas Nte 2771, Cd Universitaria, Burócrata, Culiacán 80030, Sinaloa, Mexico

**Keywords:** amaranth, germination, enzymatic hydrolysis, techno-functional properties, anti-inflammatory

## Abstract

Amaranth is a nutritional and naturally gluten-free pseudocereal with several food applications. The germination and pepsin/pancreatin hydrolysis in amaranth releases antioxidant and anti-inflammatory compounds but the hydrolysis times (270 or 360 min) are too long to scale up in the development of amaranth functional ingredients. The aim of this study was to estimate the influence of the germination and pepsin/pancreatin hydrolysis reduction time on the techno-functional properties and nutraceutical potential of amaranth flours and hydrolysates. The germination process increased 12.5% soluble protein (SP), 23.7% total phenolics (TPC), 259% water solubility, and 26% oil absorption in germinated amaranth flours (GAFs) compared to ungerminated amaranth flours (UAFs). The ungerminated (UAFH) and germinated (GAFH) amaranth hydrolysates showed values of degree of hydrolysis up to 50% with 150 min of sequential (pepsin + pancreatin) hydrolysis. The enzymatic hydrolysis released 1.5-fold SP and 14-fold TPC in both amaranth flours. The water solubility was higher in both hydrolysates than in their unhydrolyzed flour counterparts. The reduction in hydrolysis time did not significantly affect the nutraceutical potential of GAFH, enhancing its potential for further investigations. Finally, combining germination and enzymatic hydrolysis in amaranth enhances nutraceutical and techno-functional properties, increasing the seed. Consequently, GAF or GAFH could be used to elaborate on functional or gluten-free food products.

## 1. Introduction

Amaranth (*Amaranthus hypochondriacus*) is an important ancestral crop in Mexico with important agronomical features and great nutritional value [1]. Amaranth seed is a good source of quality protein, minerals, vitamins, and bioactive compounds [1,2,3]. Celiac disease is characterized by an inflammation in the small intestine triggered by gluten proteins from wheat, barley, and rye [4]. Amaranth is a gluten-free grain, and it seems to be a great substitute for wheat in bread, cookies, and other food products [5,6,7]. Moreover, the potential anti-inflammatory activity from amaranth ingredients has been previously reported [2,8].

Diverse amaranth food products with nutritional quality, nutraceutical potential, and good sensorial characteristics have been developed, using technologies such as extrusion, germination, roasting, and enzymatic hydrolysis [5,6,7,9]. Amaranth germination improves its nutritional and nutraceutical value [10,11]. This process can cause changes in chemical composition, nutritional quality, and grain structure, leading to changes in the techno-functional properties and food application [12]. Additionally, the production and application of germinated seed flours is an innovative area in the food industry for the development of healthy products [5,13]. Moreover, enzymatic hydrolysis has been used for the release of bioactive peptides [2,8] or in vegetal protein to improve techno-functional properties, as solubility and gelling, that determine its food application [14].

The combination of germination and enzymatic hydrolysis can release phytochemicals as phenolic compounds and peptides with potential antioxidant and anti-inflammatory biological activities. However, the hydrolysis times (270 or 360 min) applied were too long and not practical to be used in the industry to generate amaranth functional ingredients [2,10]. Moreover, some techno-functional properties in raw/germinated amaranth flours have been evaluated [12,15,16] but not in amaranth hydrolysates as in other seeds hydrolysates [17,18]; several investigations focus only on the biological potential. Therefore, the aim of this study was to estimate the influence of germination and pepsin/pancreatin hydrolysis time reduction on the techno-functional properties and nutraceutical potential of amaranth flours and hydrolysates. So, the hypothesis of this study is that the hydrolysis time reduction did not significantly affect the nutraceutical potential of amaranth hydrolysates.

## 2. Materials and Methods

### 2.1. Materials

Amaranth seeds (*Amaranthus hypochondriacus*) were grown and harvested in 2016 in Temoac, Morelos, Mexico. The seeds were cleaned and stored in containers at 4 °C until analysis.

### 2.2. Amaranth Germination

The germinated amaranth flour (GAF) was achieved as previously reported [10]. The germination process was accomplished in a light/dark photoperiod (12/12 h) at 30 °C for 78 h with 80% relative humidity. The amaranth sprouts were freeze-dried. The ungerminated and germinated material was subjected to milling in a Cyclone Sample Mill (UD Corp, Boulder, CO, USA). The amaranth flours were packed in polyethylene bags and stored at 4 °C until analysis.

### 2.3. Enzymatic Hydrolysis

In vitro simulated protein digestion was achieved as previously reported [10], with a modification in the hydrolysis time to obtain hydrolysates with a degree of hydrolysis up to 50%. Briefly, amaranth flours were dissolved in water to acquire a 10% solution (*w*/*v*). The in vitro digestion was conducted first with pepsin (90 min) at an enzyme/substrate ratio of 1:20 (37 °C, pH 2.0). Afterward, pancreatin (1:20) was added at 37 °C and pH 7.5 for 60 min. The hydrolysates were heated at 75 °C for 20 min to inactivate the enzyme. The solutions were centrifuged at 20,000× *g* for 15 min at 4 °C. The supernatant was then collected, freeze-dried, and stored at −20 °C until analysis.

### 2.4. Degree of Hydrolysis (DH)

The DH was determined by measuring the total nitrogen content (N) soluble in 10% trichloroacetic acid (TCA) [19]. Briefly, 0.2 g of hydrolysate was mixed with 30 mL of 10% TCA in water. Then, the sample was homogenized and centrifuged at 10,000 rpm for 20 min at 10 °C. The supernatant was recovered, and the soluble N in the TCA solution was determined by the Kjeldahl method. The degree of hydrolysis was calculated by the formula:

DH = ((10% TCA − soluble N in sample)/Total N in sample) × 100

### 2.5. Soluble Protein Quantification

Soluble protein was determined with the DC Protein Assay (Biorad, Hercules, CA, USA). Briefly, diluted samples (1:50) were set in a 96-well plate, and mixed with reagent A + reagent B, agitated, and then incubated for 15 min/RT. The absorbance was read at 630 nm in a Microplate Reader (Biotek Instruments, Winooski, VT, USA). The protein concentration (mg/g) was calculated using a bovine serum albumin (BSA) standard curve (0.01 to 1.5 mg/mL).

### 2.6. SDS-PAGE

Protein bands of flours and hydrolysates of amaranth samples were analyzed using a Mini-Protean Tetra Cell (Bio-Rad Laboratories Inc., Hercules, CA, USA). Gels consisted of a 15% polyacrylamide resolving gel (pH 8.8) and a 5% stacking gel (pH 6.8). Samples of amaranth were diluted with Laemmli Sample Buffer (Bio-Rad Laboratories) with β-mercaptoethanol and boiled for 5 min prior to loading [9]. A prestained precision protein marker, Plus Protein Dual Color standard (Bio-Rad, Hercules, CA, USA) was used. The electrophoresis in tris-glycine buffer was achieved for 120 min at 110 volts. Subsequently, the gels were fixed and stained with Coomassie Blue G-250. Gels images were analyzed using Gel Doc™ XR + Gel Documentation System of Bio-Rad.

### 2.7. Total Phenolic Compounds (TPCs)

The TPC in amaranth samples was extracted and determined as previously reported [2]. The samples were dissolved in 80% methanol and sonicated for 15 min at room temperature, then centrifuged at 4 °C, at 10,000 rpm for 10 min. The supernatant obtained was collected and the same procedure was repeated for the remaining pellet. The supernatant was dried in a concentrator and stored at 4 °C until further use. TPC was determined using the Folin-Ciocalteau colorimetric method. The curve/sample was mixed with the Folin−Ciocalteu 2N reagent and after 5 min a 2N sodium carbonate solution was added. After that, distilled water was added to each tube. The sample was incubated at room temperature for 90 min and read in a 96-well plate at 740 nm. Quantification was carried out using a gallic acid curve and the results were expressed as mg of gallic acid equivalents (mg GAE) per 100 g of sample.

### 2.8. ORAC

The antioxidant capacity of the amaranth flours and hydrolysates was determined using the oxygen radical absorbance capacity (ORAC) assay, according to the method previously described [2]. The amaranth samples (dissolved in PBS) were used for antioxidant capacity, using fluorescein (substrate) and trolox (standard). AAPH (2,2′-Azobis(2-methylpropionamidine) dihydrochloride) was used as a peroxyl generator. The samples were placed in a microplate reader (Bio-Tek Instruments, Winooski, VT, USA), with an excitation of 485 nm and a filter emission of 520 nm, and absorbances were monitored every 2 min for 2 h. The results were calculated based on the differences in area under the fluorescent falling curve between the blank and the samples and were expressed as millimoles of trolox equivalents (mmol of ET)/100 g.

### 2.9. Anti-Inflammatory Activity

Anti-inflammatory activity was investigated through the determination of inhibition of nitric oxide (NO) using an inflammation-activated RAW 264.7 cell system [2]. A murine macrophage RAW 264.7 (American Type Culture Collection, Manassas, VA, USA) cell line was cultured in DMEM (High-glucose Dulbecco’s Modified Eagle’s Medium) growth medium supplemented with 10% FBS (Fetal bovine serum, Biowest, Mexico) and 1% penicillin/streptomycin (Thermo Fisher Scientific, Grand Island, NY, USA). The cells were maintained in a humidified incubator (37 °C and 5% CO_2_ atmosphere). Macrophages were seeded in 96-well plates at a density of 5 × 10^4^ cells/well and allowed to grow to confluence overnight. The cells were pre-treated (24 h) with amaranth hydrolysates (1 mg/mL) dissolved in serum-free medium, then elicited (24 h) with lipopolysaccharide (LPS, from *Escherichia coli* O55:B5) at 1 µg/mL. After LPS elicitation, the medium was removed (for NO production), and cell viability was determined using a Cell Titer 96^®^ Aqueous One Solution Proliferation Assay kit (Promega, Madison, WI, USA). The nitrite concentrations (NO production) were measured in the macrophages culture medium by a Griess reaction. Briefly, 100 μL of medium were plated in a 96-well plate and an equal amount of the Griess reagent (1% (*w*/*v*) sulfanilamide and 0.1% (*w*/*v*) N-1-(naphthyl) ethylenediamine-diHCl in 2.5% (*v*/*v*) H_3_PO_4_) was added. After 15 min, the absorbance was measured at 550 nm in a Synergy MX microplate reader (BioTek Instruments, Winooski, VT, USA). The amount of NO was calculated using a sodium nitrite standard curve (0–10 µg/mL). All experiments were performed in three independent trials with three replicates per trial.

### 2.10. Techno-Functional Properties

#### 2.10.1. Water Absorption Index (WAI) and Water Solubility Index (WSI)

The WAI and WSI were determined according to the methodology previously reported [20]. First, sample dilutions (1:12) in distilled water were placed in a 50 mL centrifuge tube and shaken lightly. The tubes were placed in a water bath at 70 °C for 30 min, and then centrifuged at 3000× *g* for 10 min. The supernatants were decanted in a pre-weighed glass vial to determine their solids after placing tubes overnight in an oven set at 105 °C. The WAI and the WSI were calculated according to the following formulas:

WAI (g/g) = Sediment weight/sample weight

WSI (%) = (weight of the dry solids in the supernatant/weight of the sample) × 100

#### 2.10.2. Oil Absorption Index (OAI)

The OAI in flours/hydrolysates was determined as reported previously [21] and calculated according to the following formula:

OAI (mL/g) = mL of oil absorbed/g of flour/hydrolysate

#### 2.10.3. Emulsion Activity Index (EAI)

The EAI was determined according to the turbidimetric method [22]. The oil-in-water emulsions were prepared by dispersing 25% (*v*/*v*) canola oil in a phosphate buffer (100 mM, pH 7) with 1% (*w*/*v*) protein from amaranth samples. The emulsions were homogenized at 14,000 rpm for 1 min at room temperature. For the EAI determination, 100 μL were added to 3 mL of 0.1% (*w*/*v*) sodium dodecyl sulfate (SDS) and mixed. The absorbance of the diluted emulsion was measured at 500 nm. The EAI was calculated with the following equations:

T = 2303 × (A/L) × D

EAI (m^2^/g) = (2 × T)/(Ø × C × 1000)

T = is the turbidity of the emulsion in m, A = is the absorbance at 500 nm of the diluted emulsion, D = is the dilution factor, L = is the width of the cell in m, EAI = is the emulsion activity index expressed in m^2^/g, Ø = is the volume fraction of oil, and C = is the concentration of the protein dispersion (mg/mL).

#### 2.10.4. pH

The pH was determined using a potentiometer as proposed by Espinosa-Ramírez and Serna-Saldívar, 2016 [23]. A dispersion of 0.3 g of flour/hydrolysate was carried out in 7.5 mL of distilled water and incubated for 30 min at 25 °C with continuous agitation. Subsequently, the pH was measured using a potentiometer.

### 2.11. Statistical Analysis

Data were analyzed using one-way analysis of variance, followed by a Tukey’s test to compare means (*p* < 0.05). Statistical analyses were conducted using JMP 18 software from the SAS institute (Cary, NC, USA). All analyses were conducted in at least three or six independent replicates.

## 3. Results and Discussion

### 3.1. Effect of Germination and Enzymatic Hydrolysis on the Degree of Hydrolysis and Amaranth Protein Profile

**Figure 1:**
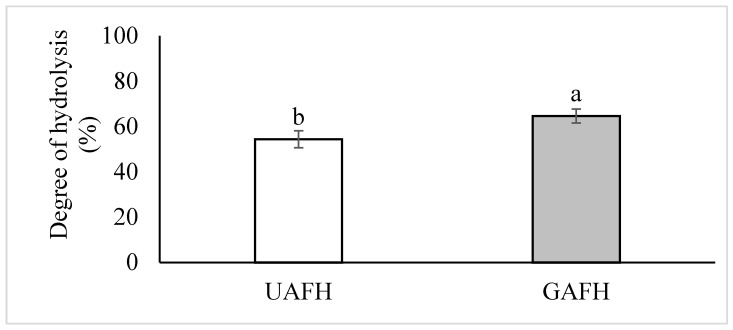
Degree of hydrolysis of ungerminated and germinated amaranth flour hydrolysates ^1^. ^1^ Pepsin (90 min) + Pancreatin (60 min). The results are means ± standard deviation of three replicates (*n* = 3). Means with different letters are significantly different (*p* < 0.0215). UAFH: ungerminated amaranth flour hydrolysate. GAFH: germinated amaranth flour hydrolysate.

The objective of this study was to estimate the influence of germination and the reduction in hydrolysis time (sequential pepsin/pancreatin hydrolysis) on the nutraceutical potential of amaranth flours and hydrolysates. Also, the techno-functional properties of amaranth flours and hydrolysates were determined. The amaranth hydrolysates were acquired using pepsin + pancreatin enzymes to simulate gastrointestinal digestion and imitate food consumption. For practical application, the hydrolysis time was reduced to 150 min of hydrolysis (90 min pepsin + 60 min pancreatin) from the 360 min [10] or 270 min [2] previous hydrolysis times. As in the case of the previous hydrolysates, it is expected to obtain amaranth hydrolysates with a DH up to 50%, and without significant losses of nutraceutical potential in the hydrolyzed sample [10]. The germinated amaranth flour hydrolysate (GAFH; 64%) showed higher values in the DH compared to ungerminated amaranth flour hydrolysates (UAFH; 54%) (Figure 1). In a previous study, amaranth flours were hydrolyzed with pepsin + pancreatin (360 min) [10]. Although there was a reduction in hydrolysis time, the DH was up to 50% in both hydrolysates.

**Figure 2:**
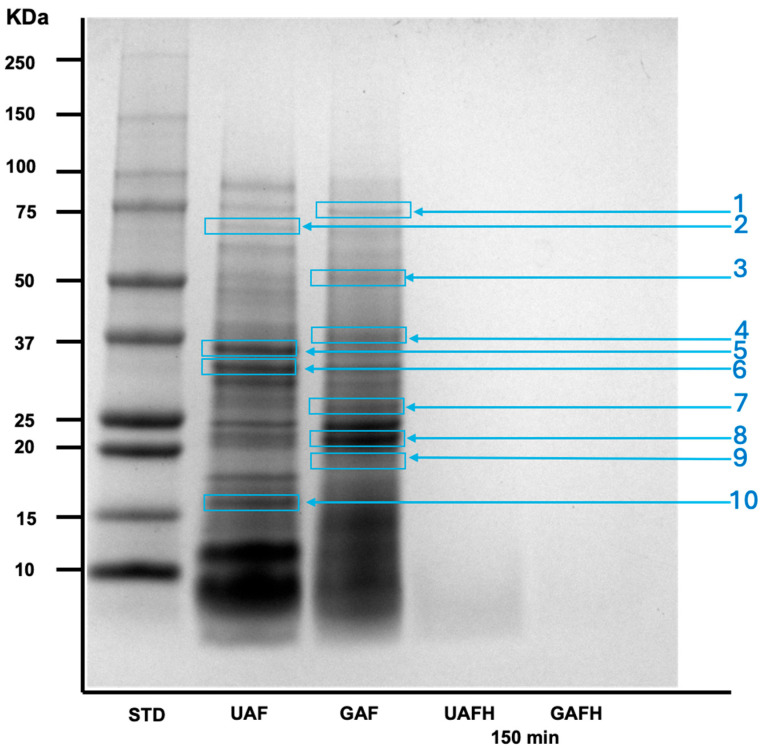
Protein profile of ungerminated/germinated amaranth flours and its pepsin/pancreatin hydrolysates. 1: Acetolactate Synthase. 2: Granule-bound starch synthase. 3: Globulin 11S. 4: Glutelin. 5: Amaranth albumin 1. 6: Globulin 7S. 7: Albumin. 8: Prosystemin. **9**: RING Zinc finger protein. 10: Superoxide dismutase [Cu-Zn]. UAF: ungerminated amaranth flour. GAF: germinated amaranth flour. UAFH: ungerminated amaranth flour hydrolysate. GAFH: germinated amaranth flour hydrolysate. STD: standard.

Figure 2 shows the protein profile of amaranth flours and its hydrolysates. The protein profile observed in the UAF and GAF (Figure 2); the protein bands were similar, as previously reported [10]. The proteins detected are acetolactate synthase, granule-bound starch synthase, globulin 11S, glutelin, globulin 7S, and superoxide dismutase [Cu-Zn], and others. Moreover, some proteins in the GAF were degraded by the germination process, as albumin and globulin. The proteins (100–10 kDa) in amaranth hydrolysates (UAFH/GAFH) were completely digested after 150 min of enzymatic treatment (90 min pepsin + 60 min pancreatin). The main objective of the SDS-PAGE (Figure 2) in this study is to verify that the SGD was effective to produce peptides (<10 kDa) from amaranth flours, as can be observed in the figure (UAFH/GAFH). We can assume that the hydrolysis time reduction did not affect the bioactive peptides generation since it is observed in Figure 2 and the DH was up to 50% as in the previous studies [10].

### 3.2. Effect of Germination and Enzymatic Hydrolysis on Bioactive Content and Nutraceutical Potential in Amaranth Flours/Hydrolysates

The TPC values were increased 23.7% (*p* < 0.05) by the germination process (Table 1), similarly as previously reported [10,24]. The germination process is known to have an increased enzymatic activity as s it facilities the increased content of bioactive compounds [25]. In the case of the phenolic compounds, this is usually related to the activation of PAL (phenylalanine ammonia-lyase) enzyme [10]. Additionally, the SP content was increased by germination but in lower values than previously reported (12% vs. 35%) [10]. These changes are explained by the protein synthesis/proteolysis effect. The endopeptidases and proteases are usually activated in the initial phases of germination. These can degrade proteins or promote the synthesis of new proteins [26]. The antioxidant potential (Aox) in the amaranth flours and hydrolysates is depicted in Table 1. The antioxidant potential was better in the GAF compared to the UAF, as formerly detected [10], presumably as a result of the increase in TPC and SP.

The enzymatic (pepsin/pancreatin) hydrolysis significantly increased TPC values (14-fold) in both amaranth hydrolysates compared with their unhydrolyzed counterparts. Despite the hydrolysis time reduction, the TPC values were close to those previously reported [2]. Phenolic compounds are released from macromolecules through enzymatic hydrolysis, and are correlated with the phenolic profile in the samples [10]. After enzymatic hydrolysis, the SP increased up to 50% compared to its unhydrolyzed counterparts. These results were similar to those previously reported in the GAFH with higher hydrolysis times of 205 min (180 min pepsin/25 min pancreatin) [10] and 270 (180 min pepsin/90 min pancreatin) [2]. Montoya-Rodriguez et al. (2014) [8] reported an increment in SP after enzymatic hydrolysis, which is associated with the production of small peptides (low molecular mass). In this study, peptides were not analyzed; however, as can be observed in Figure 2, after 150 min of hydrolysis, there were no observed proteins higher than 10 kDa, so small peptides may be generated.

The Aox in both hydrolysates was higher than their unhydrolyzed counterparts. The release of bioactive compounds by pepsin/pancreatin hydrolysis and germination may increase the Aox [2,10]. Although the Aox in 270 min GAFH showed higher values (74 mmol TE/100 g) [2] than the values found herein at 150 min GAFH, it still had good Aox. In a previous study, the peptide sequences (ISYNY, GRFREF, DIFAM, RFQDDQHQ, PQQEHSGEHQ, and SEPFG) from germinated amaranth were detected and may contribute to the Aox of amaranth hydrolysates [2]. Moreover, the potential anti-inflammatory activity (AIA) was evaluated as the % of nitric oxide (NO) production inhibition. NO is a molecular biomarker associated with inflammation triggering. The large amounts of biomarkers can lead to tissue damage, organ dysfunction, or tumorigenesis [27]. The amaranth hydrolysates (1 mg/mL) showed a viability higher than 85% with test control in RAW264.7 macrophages. The potential AIA in amaranth hydrolysates was assayed as the % of NO production inhibition (Table 1). The AIA values in GAFH (44.29%) and UAFH (41.63%) did not show significant differences. Interestingly, the reduction in hydrolysis time for GAFH (150 min) preparation did not affect the potential AIA. Similar AIA values (42%) were reported for germinated amaranth flours hydrolyzed for 270 min (180 min pepsin + 90 min pancreatin) [2]. The peptide sequences (QDMK, RFQDQHQ, AITGQVPRR, PQQEHSGEHQ, and HGSEPGGPR) in germinated and extruded amaranth have been reported as being responsible for the anti-inflammatory effect [2,8]. The potential of AIA of amaranth flours improves the suitability of food products for celiac patients, where inflammation is involved in the disease progression, along with gluten-free characteristics of amaranth grains [28].

### 3.3. Effect of Germination and Enzymatic Hydrolysis on the Techno-Functional Properties in Amaranth Flours/Hydrolysates

The germination process caused a decrease of 31% in the WAI (*p* < 0.0001) in amaranth flours (Table 2). The WAI is usually related to water interactions with protein and starch, and its reduction in the germination process can be attributed to the partial hydrolysis of starch and proteins [17,20]. Furthermore, WAI reduction is associated with the migration of germination products to the amorphous region in the starch granule, avoiding the hydration capacity of starch granules [29]. Also, the WSI value increased 3.6 times (*p* < 0.0001) by the germination process in amaranth flours (Table 2). The amaranth processing has shown an improvement in WSI values in roasted, extruded, and germinated flours [5,9]. The changes in WAI and WSI values may be due to the increase in the presence of soluble polysaccharides and small compounds generated during germination [5,20]. The OAI increased (26%) in GAF compared to UAF (Table 2) by germination (*p* < 0.0012). Similar changes have been reported in raw and germinated amaranth [5,15,16] and quinoa [30]. High OAI values are desirable to improve taste and palatability, as well as to increase shelf life, particularly in bakery or meat products, in which oil absorption is desired [18,30]. Furthermore, oil acts as a flavor retainer and enhances the mouth feel of food products [12]. The amaranth flours (UAF and GAF) did not show differences in EAI values. In contrast, in the literature there has been reported an increase in emulsion properties by germination in amaranth, wheat, and rice [12,31]. Moreover, the amaranth germination decreased by 13% (*p* < 0.0001) in pH values. The pH values were similar as previously reported in amaranth flours [9]. The pH is an important parameter that is associated with protein solubility and the related techno-functional properties as a water absorption capacity, emulsification, and foam formation [32].

The enzymatic hydrolysis (150 min) with pepsin + pancreatin decreased the WAI value in 91% (UAFH) and 81% (GAFH) (Table 2) (*p* < 0.0001) compared with its unhydrolyzed counterparts. Similar effects were reported in soy–maize hydrolysates [18]. The WAI reduction could be due to the production of peptides with a small molecular mass or free amino acids that cannot bind water molecules. Additionally, this is caused by the disruption of the protein network by proteases [18]. The protein WAI function is correlated with various factors, such as size, shape, the hydrophilic–hydrophobic balance of amino acids in the protein molecules, degree of hydrolysis, and the presence of lipids or carbohydrates associated with proteins [33]. WSI values increased significantly in UAFH (83%) and GAFH (49%) after enzymatic hydrolysis compared with its unhydrolyzed counterparts. Soria-Hernández et al. (2015) [18] found a significant increase (49–60%) in WSI values by hydrolyzing soy–maize concentrates. WSI is an important techno-functional property because it depends on the ability of proteins to interact with water and this affects other functional characteristics such as emulsion properties and foam formation [18]. The increase in solubility in hydrolysates is often associated with the release of peptides from the origin source [34]. A different pattern was found in the samples after enzymatic hydrolysis; the OAI values in UAFH increased by 15% (*p* < 0.0006) and in GAFH reduced by 45% (*p* < 0.0003) compared with its unhydrolyzed counterparts. In soy–maize hydrolysates, a 20% increase was reported after hydrolysis [18]. This effect is attributed to the enzymatic hydrolysis, which exposed the hydrophobic groups which allowed the physical trapping of the oil [14]. The enzymatic hydrolysis decreased EAI values by 28% (UAFH) (*p* < 0.0003) and 29% (GAFH) (*p* < 0.005) compared with its unhydrolyzed counterparts. Similar results have been found in soy-maize hydrolysates [18]. The small peptides present in hydrolysates do not form stable films close to oil droplets and cannot stabilize emulsions due to a charge repulsion that made it difficult to agglomerate and produce a globular fat membrane [35]. Moreover, UAFH and GAFH were adjusted to pH 4 as steps for enzymatic inactivation and its pH values were 4.06 (UAFH) and 4.03 (GAFH). The amaranth proteins have a low solubility at a pH close to the isoelectric point (4.5); however, in both hydrolysates an improved WSI value was observed. A similar tendency was also observed in the soy protein in acid pH condition [36]. The good solubility of amaranth hydrolysates can expand the use of products with acid pH.

## 4. Conclusions

The amaranth germinated flour (GAF) showed a higher bioactive compound content (SP/TPC), Aox, solubility and oil absorption than the UAF. The reduction in hydrolysis time (150 min) did not significantly affect the nutraceutical potential of GAFH, making it feasible for further industrial application. Combining germination and enzymatic hydrolysis (pepsin/pancreatin) enhances the nutraceutical profile of GAFH and releases bioactive compounds with antioxidant and anti-inflammatory potential. Also, the amaranth hydrolysates showed a good solubility at acid pH. GAFH processing increased seed value and generated an innovative plant-based ingredient with several potentials in e food development. Therefore, GAF or GAFH could be used to develop o functional or gluten-free food products.

## Figures and Tables

**Table 1:** Effect of germination and enzymatic hydrolysis on soluble protein, anti-inflammatory activity, total phenolic content, and antioxidant potential of amaranth flours/hydrolysates.

Parameter		Flour (F)	Hydrolysate ^1^ (FH)	
SP (mg/g)	UA	82.47 ± 0.76 ^B^_b_	131.52 ± 8.96 ^A^_a_	(*p* < 0.0007)
	GA	92.80 ± 1.32 ^B^_a_	144.68 ± 3.40 ^A^_a_	(*p* < 0.0001)
		(*p* < 0.0003)	NS	
TPC (mg GAE/100 g)	UA	21.22 ± 1.14 ^B^_b_	301.69 ± 1.23 ^A^_b_	(*p* < 0.0001)
	GA	26.25 ± 0.75 ^B^_a_	388.64 ± 6.21 ^A^_a_	(*p* < 0.0001)
		(*p* < 0.0031)	(*p* < 0.0001)	
Aox (mmol TE/100 g)	UA	12.5 ± 0.2 ^B^_b_	38.93 ± 0.58 ^A^_a_	(*p* < 0.0001)
	GA	34.6 ± 0.4 ^B^_a_	43.20 ± 2.54 ^A^_a_	(*p* < 0.0092)
		(*p* < 0.0001)	NS	
AIA (% NOI)	UA	ND	41.63 ± 0.84 _a_	
	GA	ND	44.29 ± 1.69 _a_	
			NS	

^1^ Pepsin (90 min) + Pancreatin (60 min). The results are means ± standard deviation of six replicates (*n* = 6). Means with different capital letters in the same row are statistically different (*p* < 0.05) (Enzymatic hydrolysis effect). Different lowercase letters per column for each parameter indicate significant difference (*p* < 0.05) (Germination effect). ND. Not determined. % NOI: Percentage of nitric oxide inhibition with 1 mg/mL of LPS (Lipopolysaccharide). SP: soluble protein TPC: total phenolic content ORAC: AIA: anti-inflammatory activity. Aox: Antioxidant potential. UAF: ungerminated amaranth flour. GAF: germinated amaranth flour. UAFH: ungerminated amaranth flour hydrolysate. GAFH: germinated amaranth flour hydrolysate. NS. Not statistically significant.

**Table 2:** Effect of germination and enzymatic hydrolysis on techno-functional properties of amaranth flours.

Parameter		Flour (F)	Hydrolysate ^1^ (FH)	
WAI (g/g)	UA	5.47 ± 0.05 ^A^_a_	0.47 ± 0.02 ^B^_b_	(*p* < 0.0001)
	GA	3.77 ± 0.05 ^A^_b_	0.70 ± 0.03 ^B^_a_	(*p* < 0.0001)
		(*p* < 0.0001)	(*p* < 0.0004)	
WSI (%)	UA	13.60 ± 0.46 ^B^_b_	97.47 ± 0.60 ^A^_a_	(*p* < 0.0001)
	GA	48.90 ± 2.15 ^B^_a_	98.28 ± 0.39 ^A^_a_	(*p* < 0.0001)
		(*p* < 0.0001)	NS	
OAI (mL/g)	UA	1.23 ± 0.03 ^B^_b_	1.41 ± 0.01 ^A^_a_	(*p* < 0.0006)
	GA	1.55 ± 0.06 ^A^_a_	0.86 ± 0.08 ^B^_b_	(*p* < 0.0003)
		(*p* < 0.0012)	(*p* < 0.0003)	
EAI (m^2^/g)	UA	2.24 ± 0.08 ^A^_a_	1.62 ± 0.04 ^B^_a_	(*p* < 0.0003)
	GA	2.38 ± 0.21 ^A^_a_	1.69 ± 0.04 ^B^_a_	(*p* < 0.005)
		NS	NS	
pH	UA	6.56 ± 0.00 ^A^_a_	4.03 ± 0.00 ^B^_b_	(*p* < 0.0001)
	GA	5.72 ± 0.06 ^A^_b_	4.06 ± 0.00 ^B^_a_	(*p* < 0.0001)
		(*p* < 0.0001)	(*p* < 0.0001)	

^1^ Pepsin (90 min) + Pancreatin (60 min). The results are means ± standard deviation of three replicates (*n* = 3). Means with different capital letters in the same row are statistically different (*p* < 0.05) (enzymatic hydrolysis effect). Different lowercase letters per column for each parameter indicate significant difference (*p* < 0.05) (germination effect). WAI: Water Absorption Index. WSI: Water Solubility Index. OAI: Oil Absorption Index. EAI: Emulsion Activity Index. UAF: ungerminated amaranth flour. GAF: germinated amaranth flour. UAFH: ungerminated amaranth flour hydrolysate. GAFH: germinated amaranth flour hydrolysate. NS. Not statistically significant.

## Data Availability

Data available on request due to restrictions.

## References

[1] Montoya-Rodríguez A., Gómez-Favela M.A., Reyes-Moreno C., Milán-Carrillo J., González de Mejía E. (2015). Identification of Bioactive Peptide Sequences from Amaranth (*Amaranthus hypochondriacus*) Seed Proteins and Their Potential Role in the Prevention of Chronic Diseases. Compr. Rev. Food Sci. Food Saf..

[2] Sandoval-Sicairos E.S., Milán-Noris A.K., Luna-Vital D.A., Milán-Carrillo J., Montoya-Rodríguez A. (2021). Anti-inflammatory and antioxidant effects of peptides released from germinated amaranth during in vitro simulated gastrointestinal digestion. Food Chem..

[3] Balakrishnan G., Schneider R.G. (2022). The Role of Amaranth, Quinoa, and Millets for the Development of Healthy, Sustainable Food Products–A *Concise* Review. Foods.

[4] Tye-Din J.A., Galipeau H.J., Agardh D. (2018). Celiac Disease: A Review of Current Concepts in Pathogenesis, Prevention, and Novel Therapies. Front. Pediatr..

[5] Chauhan A., Saxena D.C., Singh S. (2015). Total dietary fibre and antioxidant activity of gluten free cookies made from raw and germinated amaranth (*Amaranthus* spp.) flour. LWT-Food Sci. Technol..

[6] Carmona-Garcia R., Agama-Acevedo E., Pacheco-Vargas G., Bello-Perez L.A., Tovar J., Alvarez-Ramirez J. (2022). Pregelatinised amaranth flour as an ingredient for low-fat gluten-free cakes. Int. J. Food Sci. Technol..

[7] Yeşil S., Levent H. (2022). The influence of fermented buckwheat, quinoa and amaranth flour on gluten-free bread quality. LWT.

[8] Montoya-Rodríguez A., de Mejía E.G., Dia V.P., Reyes-Moreno C., Milán-Carrillo J. (2014). Extrusion improved the anti-inflammatory effect of amaranth (*Amaranthus hypochondriacus*) hydrolysates in LPS-induced human THP-1 macrophage-like and mouse RAW 264.7 macrophages by preventing activation of NF-κB signaling. Mol. Nutr. Food Res..

[9] Milan-Carrillo J., Montoya-Rodríguez A., Reyes Moreno C. (2012). High-Antioxidant Capacity Beverages Based on Extruded and Roasted Amaranth (*Amaranthus hypochondriacus*) Flour. ACS Symp. Ser..

[10] Sandoval-Sicairos E.S., Domínguez-Rodríguez M., Montoya-Rodríguez A., Milán-Noris A.K., Reyes-Moreno C., Milán-Carrillo J. (2020). Phytochemical Compounds and Antioxidant Activity Modified by Germination and Hydrolysis in Mexican Amaranth. Plant Foods Hum. Nutr..

[11] Pilco-Quesada S., Tian Y., Yang B., Repo-Carrasco-Valencia R., Suomela J.-P. (2020). Effects of germination and kilning on the phenolic compounds and nutritional properties of quinoa (*Chenopodium quinoa*) and kiwicha (*Amaranthus caudatus*). J. Cereal Sci..

[12] Gupta A., Sharama S., Singh B. Influence of Germination Conditions on the Techno-functional Properties of Amaranth flour. Proceedings of the International Conference of Food Propierties (ICFP 2018).

[13] Cornejo F., Novillo G., Villacrés E., Rosell C.M. (2019). Evaluation of the physicochemical and nutritional changes in two amaranth species (*Amaranthus quitensis* and *Amaranthus caudatus*) after germination. Food Res. Int..

[14] Nisov A., Ercili-Cura D., Nordlund E. (2020). Limited hydrolysis of rice endosperm protein for improved techno-functional properties. Food Chem..

[15] Alarcón-García M.A., Perez-Alvarez J.A., López-Vargas J.H., Pagán-Moreno M.J. (2021). Techno-Functional Properties of New Andean Ingredients: Maca (*Lepidium meyenii*) and Amaranth (*Amaranthus caudatus*). Proceedings.

[16] Badia-Olmos C., Laguna L., Haros C.M., Tárrega A. (2023). Techno-Functional and Rheological Properties of Alternative Plant-Based Flours. Foods.

[17] Gamel T.H., Linssen J.P., Mesallam A.S., Damir A.A., Shekib L.A. (2006). Seed treatments affect functional and antinutritional properties of amaranth flours. J. Sci. Food Agric..

[18] Soria-Hernández C., Serna-Saldívar S., Chuck-Hernández C. (2015). Physicochemical and Functional Properties of Vegetable and Cereal Proteins as Potential Sources of Novel Food Ingredients. Food Technol. Biotechnol..

[19] Shannon E., Hayes M. (2025). Alaria esculenta, Ulva lactuca, and Palmaria palmata as Potential Functional Food Ingredients for the Management of Metabolic Syndrome. Foods.

[20] Du S.-K., Jiang H., Yu X., Jane J.-L. (2014). Physicochemical and functional properties of whole legume flour. LWT-Food Sci. Technol..

[21] Paredes-López O., Ordorica-Falomir C., Olivares-Vázquez M.R. (1991). Chickpea Protein Isolates: Physicochemical, Functional and Nutritional Characterization. J. Food Sci..

[22] Chen B., Pangloli P., Dia V.P. (2024). Comparative Physicochemical and Functional Analyses of Protein Ingredients and Their Enzymatic Hydrolysates from Industrial Hempseed (*Cannabis sativa* L.) Hearts. ACS Food Sci. Technol..

[23] Espinosa-Ramírez J., Serna-Saldívar S.O. (2016). Functionality and characterization of kafirin-rich protein extracts from different whole and decorticated sorghum genotypes. J. Cereal Sci..

[24] Perales-Sánchez J.X.K., Reyes-Moreno C., Gómez-Favela M.A., Milán-Carrillo J., Cuevas-Rodríguez E.O., Valdez-Ortiz A., Gutiérrez-Dorado R. (2014). Increasing the Antioxidant Activity, Total Phenolic and Flavonoid Contents by Optimizing the Germination Conditions of Amaranth Seeds. Plant Foods Hum. Nutr..

[25] Guardado-Félix D., Serna-Saldivar S.O., Cuevas-Rodríguez E.O., Jacobo-Velázquez D.A., Gutiérrez-Uribe J.A. (2017). Effect of sodium selenite on isoflavonoid contents and antioxidant capacity of chickpea (*Cicer arietinum* L.) sprouts. Food Chem..

[26] de Souza Rocha T., Hernandez L.M.R., Chang Y.K., de Mejía E.G. (2014). Impact of germination and enzymatic hydrolysis of cowpea bean (*Vigna unguiculata*) on the generation of peptides capable of inhibiting dipeptidyl peptidase IV. Food Res. Int..

[27] Conforti F., Menichini F. (2011). Phenolic Compounds from Plants as Nitric Oxide Production Inhibitors. Curr. Med. Chem..

[28] de la Barca A.M.C., Rojas-Martínez M.E., Islas-Rubio A.R., Cabrera-Chávez F. (2010). Gluten-Free Breads and Cookies of Raw and Popped Amaranth Flours with Attractive Technological and Nutritional Qualities. Plant Foods Hum. Nutr..

[29] Cornejo F., Rosell C.M. (2015). Influence of germination time of brown rice in relation to flour and gluten free bread quality. J. Food Sci. Technol..

[30] Jan R., Saxena D.C., Singh S. (2017). Effect of Germination on Nutritional, Functional, Pasting, and Microstructural Properties of Chenopodium (*Chenopodium album*) Flour. J. Food Process. Preserv..

[31] Sibian M.S., Saxena D.C., Riar C.S. (2017). Effect of germination on chemical, functional and nutritional characteristics of wheat, brown rice and triticale: A comparative study. J. Sci. Food Agric..

[32] Shevkani K., Singh N., Kaur A., Rana J.C. (2014). Physicochemical, Pasting, and Functional Properties of Amaranth Seed Flours: Effects of Lipids Removal. J. Food Sci..

[33] Chavan U.D., McKenzie D.B., Shahidi F. (2001). Functional properties of protein isolates from beach pea (*Lathyrus maritimus* L.). Food Chem..

[34] McCarthy A.L., O’Callaghan Y.C., O’Brien N.M. (2013). Protein Hydrolysates from Agricultural Crops—Bioactivity and Potential for Functional Food Development. Agriculture.

[35] Aluko R.E., Monu E. (2003). Functional and Bioactive Properties of Quinoa Seed Protein Hydrolysates. J. Food Sci..

[36] Horax R., Vallecios M.S., Hettiarachchy N., Osorio L.F., Chen P. (2017). Solubility, functional properties, ACE-I inhibitory and DPPH scavenging activities of Alcalase hydrolysed soy protein hydrolysates. Int. J. Food Sci. Technol..

